# Guided Personalized Surgery (GPS) in Posterostabilized Total Knee Replacement: A Radiological Study

**DOI:** 10.3390/jcm14020429

**Published:** 2025-01-10

**Authors:** Ana de Andrés-Torán, Norma G. Padilla-Eguiluz, Pablo Hernández-Esteban, Enrique Gómez-Barrena

**Affiliations:** 1 Servicio de Cirugía Ortopédica y Traumatología, Hospital La Paz-IdiPaz, 28046 Madrid, Spain; ana.deandrest@gmail.com (A.d.A.-T.); pablo.hernandezesteban@gmail.com (P.H.-E.); 2 Departamento de Cirugía, Facultad de Medicina, Universidad Autónoma de Madrid, 28029 Madrid, Spain; norma.padilla@uam.es

**Keywords:** prosthesis, navigation, alignment, patellar height, joint line, TKR alignment precision

## Abstract

**Background**: Surgical accuracy in total knee replacement (TKR) may vary with the surgeon, the patient preoperative deformity, and the guiding system to perform the procedure. Navigation systems attempt to increase the intraoperative information the surgeon requires to make the appropriate decisions, sometimes associating cumbersome procedures and unclear effectiveness to place the implant more precisely than conventional instruments. **Methods**: We conducted a retrospective case-control study with prospective data collection of radiographic measurements (alignment, joint line and patellar height) in a sample of 100 consecutive patients receiving TKR Optetrak Logic PS, either with standard surgical technique with Trulion Instrumentation (*n* = 59) or with the Guided Personalized Surgery (GPS) system (*n* = 41). **Results**: The GPS group improved the alignment of the mechanical Lateral Distal Femoral Angle (mLDFA) in 1.6° compared to the control (*p* = 0.003), but not evident in the mechanical Medial Proximal Tibial Angle (mMPTA) (*p* = 0.132). The GPS system achieved a normal patellar height in 98% of cases, according to the Blackburne–Peel Index (BP), compared to 71% in the control group (*p* = 0.002). This was obtained in the femoral side, as measured in the Epicondylar Ratio (ER) (*p* = 0.004). A lower dispersion of postoperative measurements was observed in the GPS group in comparison with the control, being statistically significant in mMPTA (*p* = 0.000), CD-Index (*p* = 0.011), IS-Index (*p* = 0.002), mIS-Index (*p* = 0.008), BP-Index (*p* = 0.011), and ER (*p* = 0.004). **Conclusions**: Better post-surgical restoration of joint line and patellar height is observed in surgeries performed with the GPS system, as well as a tendency to more accurate mechanical alignment and lower inter-patient variability, suggesting higher reproducibility.

## 1. Introduction

Total Knee Arthroplasty (TKA) is one of the most frequent surgical interventions in reconstructive surgery, used to treat disabling pain and functional impairment caused by advanced osteoarthritis that does not respond to conservative treatments. Numerous studies estimate a significant increase in the number of TKAs over the years, which should be considered when planning patient care, as it is a major health problem [1,2,3,4].

According to a study by Nam et al., only 66% of patients who underwent TKA reported the post-surgical status of their knee as completely normal. In contrast, patients undergoing Total Hip Arthroplasty (THA) reached such results in 87% of cases [5]. The correct execution of a planned alignment of the prosthetic components implies better results in the surgical intervention [6,7], as well as adequate knee balance achieved through bone surgical resection for femorotibial gap balancing after TKA [8]. However, other authors report that there is no evidence that slight malalignment, to tolerable degrees, increases the risk of implant revision or poor functional outcomes [9].

Currently, different types of TKA alignments have been described. The Mechanical Alignment (MA) is classically advocated by many authors as the technique of choice. The joint line is perpendicular to the mechanical axis of the limb and parallel to the ground [10]. The instruments most frequently used in conventional TKA surgery allow femoral cuts to be aligned using intramedullary guides in the femur (with a variable oscillation within the canal) and extramedullary guides in the tibia. Both guides use the anatomical axis as the reference, and the surgeon’s subjective appreciation of the guide alignment is decisive. Navigated aids in surgery allow intraoperative calculation of the limb mechanical axis, based on intraoperative references.

Due to current technological and scientific advances, new surgical techniques are available that may allow a more accurate surgery in the alignment of prosthetic components and, presumably, better results in the future.

At the moment, there is no evidence indicating that all the navigation systems are equivalent in terms of outcomes [11]. Navigation-supported TKA through the Guided Personalized Surgery (GPS) system pretends to orient the guides with a portable system considered to provide real-time information during surgery, enabling a MA of the prosthetic components [12,13]. Integrated with the Exactech Optetrak Logic TKR design, the GPS system relies on surgical tracers or trackers positioned on the surface of the bone endings and the cutting guides to acquire intraoperative data of the knee anatomy, referenced to the hip center of rotation and the malleoli to estimate the mechanical axis.

Our hypothesis is that this strategy may increase the accuracy in the alignment evaluated with the positioning of the knee prosthetic components and may decrease the variability (increasing the precision) in the positioning of the knee components, independently of the surgeon or center experience.

We conducted an observational study, based on pre and postoperative radiographs, comparing the alignment and the variability in the implant positioning in patients receiving the Total Knee Replacement using the Optetrak Logic PS with the support of the GPS, controlled with patients receiving the same implant with conventional guides.

## 2. Materials and Methods

### 2.1. Study Characteristics

This retrospective study with prospectively collected data was conducted from May 2019 to December 2022, at La Paz University Hospital (HULP), Madrid, Spain, as a part of a wider study (phase IV clinical trial, code: CR05-007, Ethical approval No.: PI-5055). This study aims to compare the prosthetic alignment of patients who had undergone TKA surgery in our hospital. A total of 100 consecutive patients scheduled for surgery using the Optetrak Logic PS system, either with the conventional surgical technique with Trulion Instrumentation (Conventional group, *n* = 59) or with the Exatech^®^ Guided Personalized Surgery system (GPS group, *n* = 41), were included. The assignment of the technique to be performed was based on the system’s availability in the operating room and was unrelated to the patient, avoiding any selection bias. The patients were prospectively followed up for 3, 6, and 12 months after surgery with radiographic evaluation (lateral and weight-bearing anteroposterior views of both knees/lower limbs). The data were obtained from the electronic records in the hospital system (HCIS, Healthcare Integrated System) and radiographs in the hospital-integrated PACS (Picture Archiving and Communication System).

### 2.2. Surgical Technique

**Figure 1:**
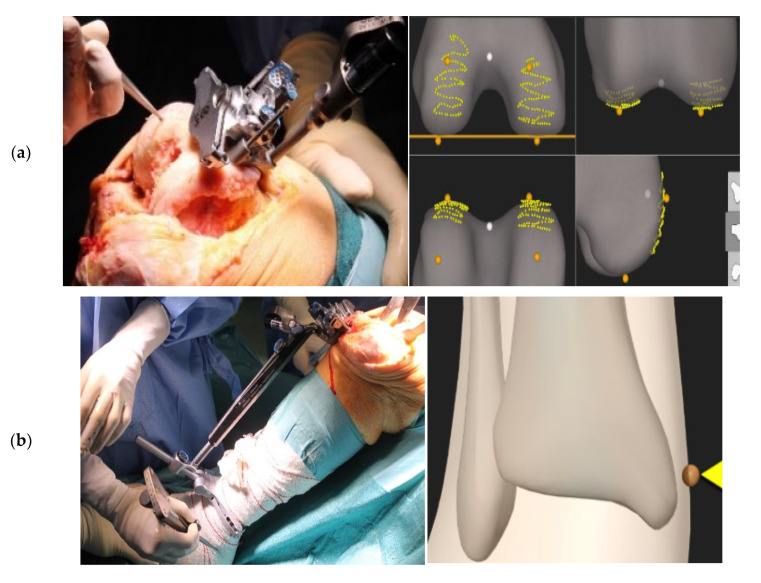
Guided Personalized Surgery system. (**a**) Acquisition of femoral landmarks. (**b**) Acquisition of tibial landmarks.

While the control knees were prepared with the Trulion^®^ Instrumentation (Exactech, Gainesville, FL, USA), the Guided Personalized System GPS (Exactech, Gainesville, FL, USA) technique was performed as follows. After confirming the hip center of rotation and the detection of the tracking instruments and pointers through the TKA-plus application, the femoral tracker was adjusted to the distal femoral resection guide and the acquisition of the femoral landmarks (posterior condyles, distal condyles) in the bone surface was performed, as shown in Figure 1. The varus/valgus and flexion/extension of the cutting guide were adjusted according to the values displayed on the screen. The distal femoral resection was verified by placing the tracker on the resected surface, completing the femoral bone preparation and positioning the component according to the technique.

For the tibial preparation, the same steps were followed: placing the tibial resection guide in a neutral position with the tracker adjusted to the guide, and using as references the tibial and peroneal malleoli, the center of the spines, the sagittal tibial plane, and the medial plateau, as shown in Figure 1. The proximal tibial resection was performed according to the tibial posterior slope and the varus/valgus values displayed on the TKA-plus screen. As for the femur, this resection was checked to place the tibial component according to the technique.

Finally, the patellar resection and the placement of the patellar prosthetic component were systematically performed following the standard technique [14].

### 2.3. Study Variables

The data were acquired from revising electronic case reports included age, sex, Body Mass Index (BMI), length of stay (days), type of system used in surgery (GPS, Conventional), post-surgical complications, and laterality (right or left knee).

The radiographic measurements were conducted by a trained investigator (AAT) following the SEROD (Spanish Knee Society) guidelines [15] and the Knee Society guidelines [16]. The pre-and post-surgery radiographic measures were obtained to assess the alignment as follows:

- The hip-knee-ankle (HKA), the mechanical lateral distal femoral angle (mLDFA), the mechanical medial proximal tibial angle (mMPTA), and the mechanical femorotibial angle (MFTA). The mLFDA and mMPTA angles were categorized as “Neutral” (mLFDA ≥ 87° and mMPTA ≥ 87°), “Varus of the tibia and femur” (mLFDA ≥ 90° and mMPTA < 87°) and “Valgus” (mLFDA < 87° and mMPTA ≥ 87°) [17,18].
- The patellar height was evaluated with the Insall–Salvati index—IS (<0.8 low, 0.8–1.2 normal, >1.2 high), Insall–Salvati modified index—ISm (>2 high, 1.2–2.1 normal), the Caton–Deschamps index—CD (<0.6 low, 0.6–1.2 normal, >1.2 high), and the Blackburne–Peel index—BP (<0.5 low, 0.5–1 normal, >1 high) [15].
- The height of the articular joint line was evaluated by the epicondylar ratio (ER) [19], and the fibular head height (FH) [20].

### 2.4. Statistical Analysis

The demographics, general characteristics, angles, and indexes data were described and analyzed by the treatment group (Conventional vs. GPS), although the angles and indexes were also longitudinally compared (pre-surgery vs. post-surgery). For continuous data, the mean was compared using the *t*-test (adjusted by equality of the variance) or the Mann–Whitney U test for non-normally distributed data. Paired data comparisons (preoperative vs. post-operative) were conducted using paired *t*-student test or the Wilcoxon signed-rank test for non-normally distributed data. The dispersion from the mean was compared by performing Bartlett’s test on the equality of variance (F) for normally distributed data and Levene’s (W0, using the median as reference) test for non-normally distributed data. The proportions were compared using Pearson’s Ji^2^ test or Fisher’s exact test if fewer than 5 cases in a cell were observed. The data were collected in Excel (Microsoft 365 MSO version 2204, Redmond, WA, USA) and analyzed using STATA 12 software (StataCorp LP, College Station, TX, USA), setting the statistical significance at 95% confidence.

## 3. Results

### 3.1. Participants Characteristics and Radiographic Assessment

#### 3.1.1. General Characteristics

Of the 100 consecutive treated patients, 60 were females and 40 were males, with a mean age of 72.5 ± 8.5 (range: 53–91) years. There were no significant differences between patients treated by conventional or GPS in terms of age, sex, or BMI, as per Table 1. No significant differences were observed either in the frequency of postoperative complications between the two groups, with three reported complications in the GPS group (persistent pain in active extension, recurrent joint effusion and acute prosthetic joint infection treated by DAIR) and three reported in the control group (prosthesis infection treated by DAIR, recurrent joint effusion and persistent lateral pain associated with iliotibial band syndrome).

#### 3.1.2. Radiographs Assessment

**Figure 2:**
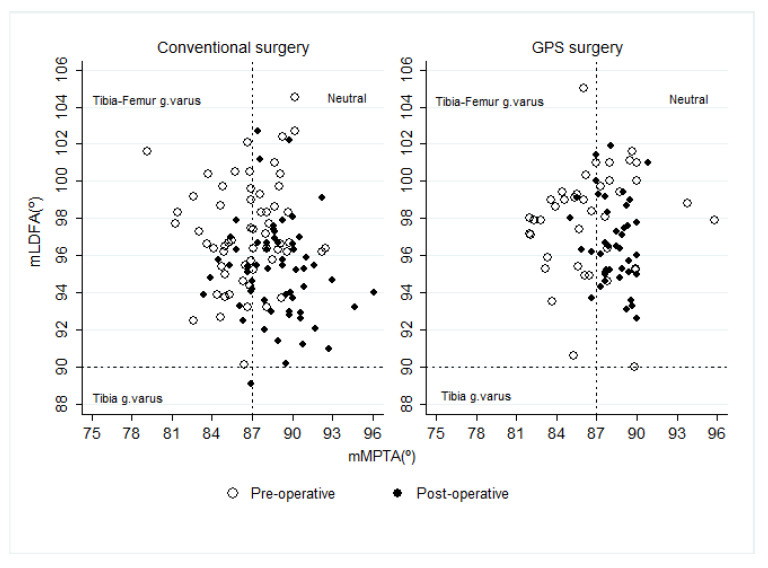
Alignment angle variations by time and type of surgery. Conventional surgery changed from 44% (*n* = 26) neutral cases preoperatively to 78% (*n* = 46) postoperatively (Pearson’s ji^2^ *p* = 0.000). GPS changed from 42% (*n* = 17) neutral preoperatively to 88% (*n* = 36) postoperatively (Pearson’s ji^2^ *p* = 0.000). Dispersion was reduced in GPS cases, with a statistically significant difference pre–post comparison in mMTPA measurements (see Table 2).

The preoperative alignment angles were similar between conventional and GPS (see Table 2). The postoperative mean mLDFA angle differed between both surgical techniques (*t*-test, *p* = 0.003), where the conventional surgery reduced it by a mean of 2.1° and GPS marginally reduced it by 1.3°. The mMPTA angle increased postoperatively by an average of 2.3° in conventional surgery and 1.7° in GPS. No mean difference was observed postoperatively between the two groups (*t*-test, *p* = 0.132), but the dispersion was higher in the conventional group (Bartlett’s test, *p* = 0.000). Combining both angles in Figure 2, we observed that 44% (*n* = 26) of the conventional surgery cases and 42% (*n* = 17) of the GPS cases were normally aligned at preoperative, which increased to 78% (*n* = 46) and 88% (*n* = 36), respectively, at postoperative. However, no differences between proportions were identified (Pearson ji^2^, *p* = 0.639).

Regarding patella height indexes, we did not find differences in the mean between the conventional and the GPS surgeries, either preoperatively or postoperatively. However, we did observe a higher dispersion of data in the conventional intervention after surgery in all the measured indexes, as per Table 3.

**Figure 3:**
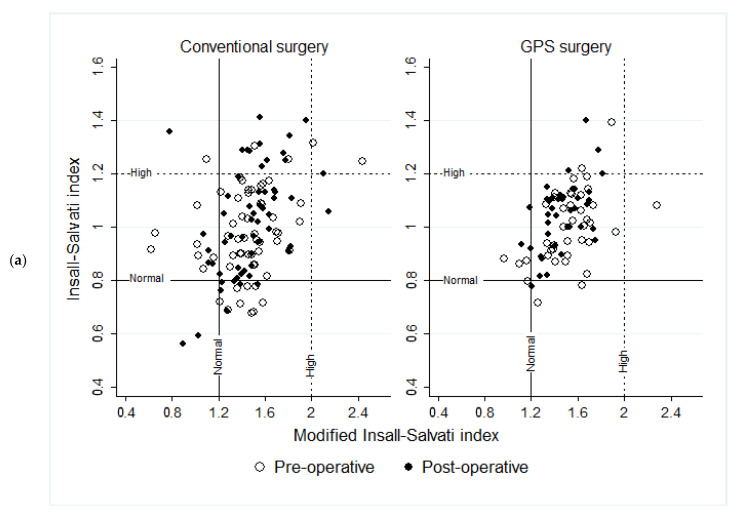

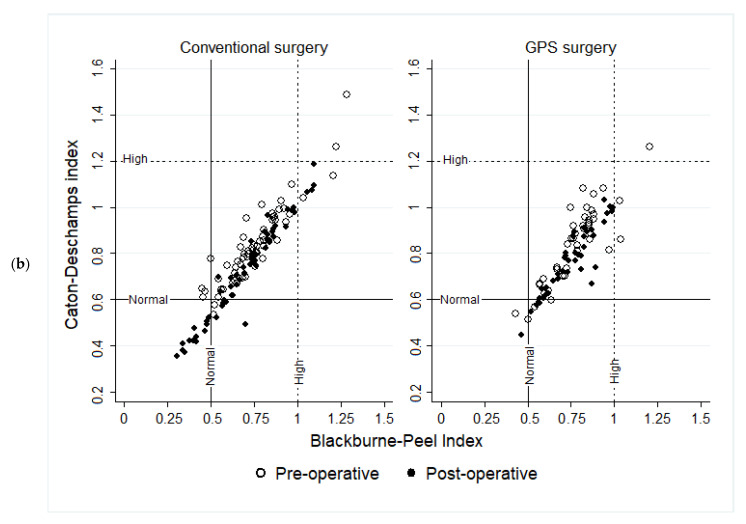
Patellar Height indexes variations by time and type or surgery. IS–Insall–Salvati index; Ism–Modified Insall–Salvati index; CD: Caton–Deschamps index; BP–Blackburne–Peel Index. (**a**) The cases were categorized as normal by the IS, and the ISm changed from pre-operative to post-operative, from 64% (n = 38) to 56% (*n* = 33) in conventional surgery (*p* = 0.215), and from 78% (n = 32) to 80% (*n* = 33) in GPS (*p* = 0.748). (**b**) The cases categorized as normal by the CD, and the BP changed from pre-operative to post-operative, from 84% (*n* = 50) to 61% (*n* = 36) in conventional surgery (*p* = 0.003), and from 82% (*n* = 34) to 88% (*n* = 36) in GPS (*p* = 0.237). All four indexes showed lower statistically significant dispersion (variance) in the postoperative GPS surgeries (see Table 3).

In the conventional surgery, the IS increased by 0.4 points, while the ISm did not change. The IS increased by 0.45 points in the GPS, but the ISm did not change. Figure 3a shows that the categorized IS did not change significantly from preoperative to postoperative, either in conventional surgery (Fisher’s exact test, *p* = 0.127) or in GPS (Fisher’s exact test, *p* = 0.530). Specifically, the categorized IS in conventional surgery changed from 76% normal to 64%, from 8% high to 22%, and from 15% low to 14%. GPS changed from 87% normal to 88%, from 5% high to 10%, and from 7% low to 2%. The categorized ISm on conventional surgery changed from 84% to 86% normal, from 2% high to 2%, and from 14% low to 12% (Fisher’s exact test, *p* = 0.999). GPS changed from 88% to 93% normal, from 2% high to 0%, and from 10% low to 7% (Fisher’s exact test, *p* = 0.712).

In conventional surgery, the CD was reduced by 0.11 points, and the BP decreased by 0.6 points. The categorized CD changed from preoperative to postoperative, from 93% normal to 67%, from 3% high to 0%, and from 3% low to 32% (Fisher’s exact test, *p* = 0.000). The categorized BP changed from 88% normal to 71%, from 7% high to 7%, and from 5% low to 22% (Fisher’s exact test, *p* = 0.024). In contraposition, the CD decreased by 0.76 points on the GPS, and the BP did not change. Figure 3b shows that the categorized indices CD and BP in the GPS did not change significantly. Specifically, the categorized CD went from 88% normal to 88%, from 2% high to 0%, and from 10% low to 12% (Fisher’s exact test, *p* = 0.574). The categorized BP went from 90% normal to 98%, 7% high to 0%, and from 2% low to 2% (Fisher’s exact test, *p* = 0.741).

**Figure 4:**
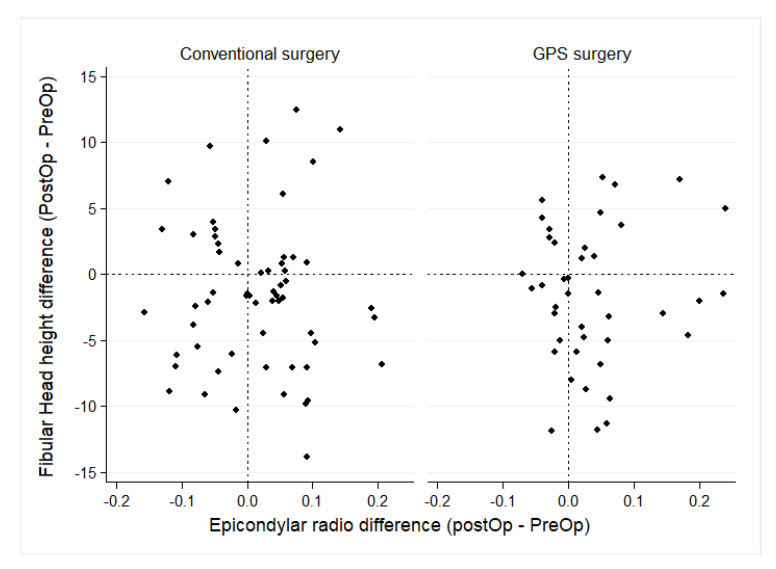
Pre-post differences in Articular Joint Line by type or surgery. The GPS achieves less dispersion in the postoperative measures of the Fibula Head height.

Finally, the epicondylar ratio (ER) did not change in the conventional surgery but increased by 0.03 points in the GPS, see Table 4. The ER showed mean and dispersion differences between the conventional and GPS interventions post-surgery. The fibula head height was reduced in both surgeries by 1.5 mm in the conventional surgery and 1.6 mm in the GPS. No FH differences were observed between the surgical technique used preoperatively and postoperatively, as per Figure 4.

## 4. Discussion

The most relevant findings of this study are based on the significant increase in the mLDFA by 1.5°, and in the ER by 0.03 mm in the GPS group with respect to the control, as well as a 13% increase in alignments considered “normal” in the mMPTA, although these differences were not statistically significant. Both groups presented an increase in the IS and a decrease in the CD post-surgery. The GPS system obtained about 98% of normopositioned patellas, according to the BP, while the control group obtained around 71%, making these differences statistically significant.

According to the first objective of the study, statistically significant differences are observed in the alignment of the prosthetic components when comparing the mean post-surgical mLDFA between the GPS group and the control group, with a difference of around 1.6° (*p* = 0.003). Although it may not be clinically relevant, since they are both within the normal ranges of femoral alignment [21], it reflects a significant improvement in the alignment of the postoperative knee. In this sense, conventional surgery is capable of obtaining neutral alignment in 78% of the cases (starting from 44% preoperatively), and GPS obtains 88% of cases with neutral alignment (starting from 42% preoperatively). In 2017, Kim et al. published a trial with a sample of 296 patients undergoing surgery for bilateral TKA at a single time, using a navigation system in one of the knees and the conventional surgical technique in the other. They found no significant differences between the two methods in pain, function, complications, or survivorship, nor were they able to find any differences in radiographic parameters of alignment [22]. In our study, we found no difference in complications but were able to find slight differences in alignment parameters. And, what seems more important, more cases in our study were neutrally aligned (as planned) with the guiding system. Furthermore, our study focused on the dispersion of the measured parameters through the variance. Other authors, such as Brin et al., concluded in a 2010 meta-analysis with 23 reviewed publications that the use of surgery guided by different navigation systems reduces outliers in the prosthesis’s mechanical axis, coronal femoral, and tibial positioning by approximately 80% [23]. The sagittal plane of TKA was not evaluated, and studies with different levels of evidence and randomization were included, so there could be overestimation biases in these results. Similar results were found in other studies [24]. As reflected in the literature, there is still a serious debate between the authors who support that navigation-guided TKA surgery allows better alignment results and those who oppose it. However, it is unclear if all navigation systems are equivalent. The limited literature on the specific navigation system analyzed in this study shows a tendency towards greater precision.

The study’s second objective addressed the comparison of the joint line height between groups. The results reflect a significant increase in the post-surgical ER in the GPS group, which confirms that the femur can be prepared to manage the joint line better. This concurs with the previously discussed results, where no patient within the GPS group showed values exceeding the limit of the normal patellar height. A higher precision of the GPS system in the distal femoral resection could explain this. If only the amount of bone that will be replaced by the prosthetic component was resected, patellar height could be further respected by preventing excessive femoral resection. Dai et al. (2019) published a retrospective study, using a database of 10,144 patients undergoing TKA using the Exactech GPS system, without comparing it with conventional instrumentation. They evaluated whether there is variability in distal femoral and proximal tibial coronal resection depending on the geographic region, the surgeon performing the procedure, their learning curve with the navigation system, and the software used. The study concluded that resection errors were not significant in any of the categories mentioned and, therefore, surgery guided by GPS navigation permits high accuracy and low variability of the outcomes of the post-surgical joint line height [25]. The importance of the joint line height and the correct femoral and tibial resection lies in the fact that if these or the ligament release are not performed as planned, the femorotibial space may be compromised in flexion and/or extension, which may result in postoperative complications [8].

As previously mentioned, the results of the patellar height in our study, according to the BP index, showed a statistically significant higher number of post-surgical patellas within the normal range in the GPS group (98%) compared to the control group (71%). In addition, there was a significant increase in the post-surgical IS in both groups, showing slightly higher patellas, always within the normal range, secondary to the reestablishment of the articular joint line. This was compensated by the lower placement of the patellar button and bigger size patellar components, with a significant decrease in the post-surgical CD in both groups, which we do not consider clinically relevant. By restoring the joint line more accurately, GPS will also lead to less dispersed post-surgical patellar height, avoiding relatively frequent complications such as persistent anterior pain and flexion limitation secondary to patella baja, or patellar instability secondary to patella alta [26,27]. These findings should be confirmed in further studies that evaluate the degree of post-surgical function and satisfaction of the patients using functional scales.

Finally, we found that one of the main advantages of GPS is the reproducibility of the results, which is related to the intraoperative control of the alignment measurements and ensuring a high concordance between the planned alignment and the postoperative result. Hannan et al., comparing the intraoperative measurements of the navigation system with the postoperative alignment in 29 patients who all underwent navigation-guided TKA with the same GPS system, similarly observed this advantage that we could prove in the comparative study with conventional instruments. High agreement was found between the intraoperative GPS and the postoperative CT measurements with a mean difference of 1.55° ± 0.22° (95% confidence interval) [28]. Similar studies support these results. Angibaud et al. found a difference equal to or less than 0.61 mm and 0.64° in resection depth and intraoperative angulation measurements, respectively, with the same navigation system used in this study and postoperatively in a series of 28 knees, concluding that it provides measurement accuracy regardless of previous deformity [29]. Consequently, although very similar component alignment outcomes are obtained in both groups in our study, a trend towards greater precision in the alignment of the components is found in the GPS navigation-guided group, with statistically significant lower dispersion of postoperative measurements compared to the control in most of the variables, such as mMPTA, CD, IS, mIS, BP, and ER. This allows higher reproducibility of the results by reducing the intraoperative subjective component, so this technique’s future seems promising and can be a beneficial tool in TKA surgeon training. Moreover, these differences between groups could be underestimated in a single-center study with a high volume of patients and a high level of experience of the team in the conventional surgical technique, so the study should be extended to other centers where TKA is not such a frequent procedure to further prove its value.

Our study has several limitations. Firstly, it uses a limited sample of 100 patients. A larger number of patients could provide more clearly significant results due to the increased sample size. Of course, a randomized, large multicentric trial, comparing different navigation systems, with outcome measurements in long-term follow-up would have been ideal, but this study still clarifies a clear trend. Second, the system was used whenever it was available, and we presume this use limited the selection bias. However, if the system was used in patients with greater preoperative deformity, the system may offer better alignment results and clinical relevance. Third, the radiographic measurements were performed by a single observer with exclusively intraobserver controls, which may lead to repeated errors in the performance of these measurements due to the lack of interobserver controls. As the evaluated parameters are part of the routine in any orthopedic department, it is felt that appropriate orthopedic training can minimize this error. Fourth, the studied radiographs were standard views in the follow-up, and some variability may apply, as not all patients were assessed in long-leg films. This might alter the alignment in some cases, providing less reliable results. Fifth, the surgical intervention was performed by different surgeons, and although they followed the same surgical protocol, slight variations in the technique may have been present. However, all participating surgeons were experienced, with more than 10 years since completing their training. Although potential discrepancies are always possible, every effort was made to minimize these through case discussions and planning.

## 5. Conclusions

Both GPS and standard TKA techniques achieved adequate alignment results. However, the study identified more precise positioning and mechanical alignment of the prosthetic components when the guiding system was used, as well as an improvement in the postoperative restoration of the patellar height and joint line. The greater reproducibility of post-surgical results in the guided group is noteworthy, ensuring more homogeneity and precision. These findings should be confirmed in subsequent studies, including those with different surgeon and team expertise, more severe patient knee deformity, and considerations of functional implications and post-surgical patient satisfaction.

## Figures and Tables

**Table 1:** Demographic and general characteristics, by type of treatment.

	Conventional Surgery (*n* = 59) Mean ± SD *n* (%)	GPS (*n* = 41) Mean ± SD *n* (%)	*p* Value
Age	72.8 ± 8.1	71.9 ± 8.9	0.588 ^t^
Female sex	36 (61.0%)	24 (58.5%)	0.803 ^j^
BMI	31.6 ± 5.1	30.4 ± 5.2	0.455 ^u^
*Surgery characteristics*			
Primary TKR (yes)	58 (98.3%)	41 (100%)	0.402 ^j^
Laterality: - Unilateral - Bilateral	39 (66.1%) 20 (33.9%)	28 (68.3%) 13 (31.7%)	0.819 ^j^

GPS–Guided Personalized Surgery; SD–Standard Deviation; BMI–Body Mass Index; TKR–Total Knee Replacement. ^t^ Student *t*-test, ^u^ Mann–Whitney test, ^j^ Pearson-Ji^2^ test.

**Table 2:** Alignment angle comparison by time and type of surgery.

Alignment Angle	Measure Time	Conventional Surgery *n* = 59 (Mean ± SD)	GPS Surgery *n* = 41 (Mean ± SD)	*Mean* *Comparison, by Surgery Type*	*Variance Comparison, by Surgery Type*
mLDFA (°)	Pre-Operative	97.2 ± 2.8	97.9 ± 2.9	*p = 0.293 ^t^*	*p = 0.819 ^t^*
	Post-Operative	95.1 ± 2.6	96.7 ± 2.3	***p = 0.003* *^t^***	*p = 0.373 ^f^*
	*Pre-Post mean comparison*	***p = 0.000*** *^pt^*	*p = 0.053 ^pt^*		
	*Pre-Post variance comparison*	*p = 0.614 ^f^*	*p = 0.150 ^f^*		
mMPTA (°)	Pre-Operative	86.6 ± 2.6	86.6 ± 3.1	*p = 0.921 ^t^*	*p = 0.233 ^f^*
	Post-Operative	88.9 ± 2.5	88.3 ± 1.3	*p = 0.132 ^t^*	***p = 0.000*** *^f^*
	*Pre-Post mean comparison*	***p* = 0.000** ^pt^	***p* = 0.002** ^pt^		
	*Pre-Post variance comparison*	*p* = 0.611 ^f^	***p* = 0.000** ^f^		
FTA (°)	Pre-Operative	175.6 ± 4.0	174.4 ± 4.1	*p = 0.128 ^z^*	*p = 0.807 ^w^*
	Post-Operative	175.5 ± 3.3	175.3 ± 2.4	*p = 0.340 ^z^*	***p = 0.049*** *^w^*
	*Pre-Post mean comparison*	*p = 0.899 ^pt^*	*p = 0.295 ^pt^*		
	*Pre-Post variance comparison*	*p = 0.179 ^w^*	***p = 0.016*** *^w^*		
HKA (°)	Pre-Operative	183.9 ± 4.1	184.4 ± 4.5	*p = 0.520 ^t^*	*p = 0.490 ^f^*
	Post-Operative	184 ± 3.4	185 ± 2.5	*p = 0.098 ^t^*	***p = 0.039 ^f^***
	*Pre-Post mean comparison*	*p = 0.834 ^pt^*	*p = 0.616 ^pt^*		
	*Pre-Post variance comparison*	*p = 0.157 ^f^*	***p = 0.001* *^f^***		

mLDFA—Mechanical Lateral Distal Femoral Angle; mMPTA—Mechanical Medial Proximal Tibial Angle; FTA—Femorotibial angle; HKA—Hip and Knee Angle; SD—Standard Deviation. Statistical tests: *^t^*—student *t*-test adjusted for homoscedasticity; *^pt^*—paired student *t*-test; *^z^*—Mann–Whitney test; *^f^*—Bartlett’s equality of variance test; *^w^*—Levene’s robust test for equality of variances using the mean. In bold, statistically significant differences.

**Table 3:** Patellar Height indexes comparison by time and type or surgery.

Index	Measure Time	Conventional Surgery *n* = 59 (Mean ± SD)	GPS Surgery *n* = 41 (Mean ± SD)	*Mean * *Comparison, by Surgery*	*Variance Comparison, by Surgery*
IS	Pre-Operative	0.98 ± 0.2	0.99 ± 0.1	*p = 0.501 ^t^*	*p = 0.237 ^f^*
	Post-Operative	1.02 ± 0.2	1.04 ± 0.1	*p = 0.431 ^t^*	***p = 0.002* *^f^***
	*Pre-Post mean comparison*	***p = 0.031* *^pt^***	***p = 0.008* *^pt^***		
	*Pre-Post variance comparison*	*p = 0.119 ^f^*	*p = 0.596 ^f^*		
ISm	Pre-Operative	1.45 ± 0.3	1.52 ± 0.2	*p = 0.157 ^z^*	*p = 0.561 ^w^*
	Post-Operative	1.48 ± 0.3	1.47 ± 0.2	*p = 0.842 ^t^*	***p = 0.008* *^f^***
	*Pre-Post mean comparison*	*p = 0.632 ^pt^*	*p = 0.199 ^pt^*		
	*Pre-Post variance comparison*	*p = 0.977 ^w^*	*p = 0.067 ^f^*		
CD	Pre-Operative	0.83 ± 0.2	0.84 ± 0.2	*p = 0.472 ^z^*	*p = 0.685 ^w^*
	Post-Operative	0.72 ± 0.2	0.77 ± 0.1	*p = 0.183 ^t^*	***p = 0.011* *^f^***
	*Pre-Post mean comparison*	***p = 0.000* *^pt^***	***p = 0.011* *^pt^***		
	*Pre-Post variance comparison*	***p = 0.023* *^w^***	*p = 0.333 ^f^*		
BP	Pre-Operative	0.75 ± 0.2	0.77 ± 0.2	*p = 0.314 ^z^*	*p = 0.575 ^w^*
	Post-Operative	0.69 ± 0.2	0.75 ± 0.1	*p = 0.099 ^t^*	***p = 0.011* *^f^***
	*Pre-Post mean comparison*	***p = 0.043* *^pt^***	*p = 0.295 ^pt^*		
	*Pre-Post variance comparison*	*p = 0.074 ^w^*	*p = 0.584 ^f^*		

IS—Insall-Salvati index; ISm—Modified Insall–Salvati index; CD—Caton–Deschamps index; BP—Blackburne–Peel Index; SD—Standard Deviation. Statistical tests: *^t^*—student *t*-test adjusted for homoscedasticity; *^pt^*—paired student *t*-test; *^z^*—Mann–Whitney test; *^f^*—Bartlett’s equality of variance test; *^w^*—Levene’s robust test for equality of variances using the mean.

**Table 4:** Articular joint line comparison by time and type of surgery.

Articular Joint Line	Measure Time	Conventional Surgery *n* = 59 (Mean ± SD)	GPS Surgery *n* = 41 (Mean ± SD)	*Mean * *Comparison, by Surgery*	*Variance Comparison, by Surgery*
ER	Pre-Operative	0.62 ± 0.1	0.63 ± 0.1	*p = 0.378 ^z^*	*p = 0.450 ^w^*
	Post-Operative	0.63 ± 0.1	0.66 ± 0.1	***p = 0.004* *^z^***	***p = 0.004* *^w^***
	*Pre-Post mean comparison*	*p = 0.179 ^pt^*	***p = 0.004* *^pt^***		
	*Pre-Post variance comparison*	*p = 0.289 ^f^*	*p = 0.317 ^w^*		
FH	Pre-Operative	17.6 ± 5.1	17.3 ± 5.3	*p = 0.795 ^t^*	*p = 0.791 ^f^*
	Post-Operative	16.1 ± 4.3	15.7 ± 3.8	*p = 0.634 ^t^*	*p = 0.451 ^f^*
	*Pre-Post mean comparison*	***p = 0.045* *^pt^***	***p = 0.028* *^pt^***		
	*Pre-Post variance comparison*	*p = 0.186 ^f^*	***p = 0.044* *^f^***		

ER—Epicondylar Ratio; FH—Fibular head height. Statistical tests: *^t^*—student *t*-test adjusted for homoscedasticity (equal variances); *^pt^*—paired student *t*-test; *^z^*—Mann–Whitney test; *^f^*—Bartlett’s equality of variance test; *^w^*—Levene’s robust test for equality of variances using the mean.

## Data Availability

Data are contained within the article.
